# Assessment of Knowledge of Monkeypox Viral Infection among the General Population in Saudi Arabia

**DOI:** 10.3390/pathogens11080904

**Published:** 2022-08-11

**Authors:** Najim Z. Alshahrani, Faris Alzahrani, Abdullah M. Alarifi, Mohammed R. Algethami, Maathir Naser Alhumam, Hatim Abdullah Mohammed Ayied, Ahmed Zuhier Awan, Abdullah Faisal Almutairi, Saeed Abdullah Bamakhrama, Budur Saad Almushari, Ranjit Sah

**Affiliations:** 1 Department of Family and Community Medicine, Faculty of Medicine, University of Jeddah, Jeddah 21589, Saudi Arabia; 2 Department of Public Health, General Directorate of Health Affairs in Aseer Region, Ministry of Health, Abha 62529, Saudi Arabia; 3 Department of Public Health, College of Health Sciences, Saudi Electronic University, Riyadh 13323, Saudi Arabia; 4 Preventive Medicine and Public Health Resident, Ministry of Health, Jeddah 21589, Saudi Arabia; 5 College of Medicine, King Faisal University, Alahsa 36363, Saudi Arabia; 6 College of Medicine, King Khalid University, Abha 62529, Saudi Arabia; 7 Faculty of Medicine, King Abdulaziz University, Jeddah 21589, Saudi Arabia; 8 Khaitan Primary Healthcare Center, Khaitan 83001, Kuwait; 9 Department of Family Medicine, General Directorate of Health Affairs in Aseer Region, Ministry of Health, Abha 62529, Saudi Arabia; 10 Department of microbiology, Institute of Medicine, Tribhuvan University Teaching Hospital, Kathmandu 44600, Nepal; 11 Research Scholars, Harvard Medical School, Boston, MA 02115, USA

**Keywords:** monkeypox, monkeypox virus, knowledge, public knowledge, outbreak, Saudi Arabia

## Abstract

Monkeypox is re-emerging and spreading over the world, posing a serious threat to human life, especially in non-endemic countries, including Saudi Arabia. Due to the paucity of research on knowledge about monkeypox in Saudi Arabia, this study aimed to evaluate the general population’s knowledge of monkeypox in a sample of the country. A web-based cross-sectional survey was conducted from 25 May 2022 to 15 July 2022. Participants’ knowledge about monkeypox on a 23-item scale and socio-demographic characteristics were gathered in the survey. Pearson’s Chi-square test was used to compare knowledge level (categorized into high and low) and explanatory variables. Out of 480, only 48% of the respondents had high knowledge (mean score > 14). Participants’ age, marital status, residential region, living in the urban area, education level, employment status, being a healthcare worker, income, and smoking status were significantly associated with the level of knowledge about monkeypox (*p* < 0.01). Overall, social media (75.0%) was the most frequently reported source from where participants obtained monkeypox-related information followed by TV and radio (45.6%), family or friend (15.6%), and healthcare provider (13.8%). We found that overall knowledge of monkeypox infection was slightly poor among the Saudi population. These findings highlight the urgent need for public education on monkeypox to promote awareness and engage the public ahead of the outbreak.

## 1. Introduction

Human monkeypox is a zoonotic orthopoxvirus infection caused by the monkeypox virus (MPXV) and is most prevalent in tropical regions of Western and Central Africa [1,2]. It was not recognized as a distinct viral infection in humans until 1970, when MPXV was isolated from a patient with suspected smallpox infection in the Democratic Republic of the Congo (DRC) [3]. The monkeypox transmission route is through droplets and by contact with skin lesions or contaminated bodily fluids and materials, with an incubation period ranging from 5 to 21 days [4,5]. Moreover, monkeypox virus can be transmitted through the vertical route (i.e., mother to fetus) [6,7]. Human monkeypox is self-limiting and symptoms usually go away within 14–21 days [2,4]. Rodents are believed to be the reservoir for MPXV and contact with animals, alive or dead, and eating bush food are the risk factors. However, the reservoir animal remains to be confirmed [8]. Monkeypox symptoms range from mild to severe, characterized mainly by itchy to painful skin lesions and including fever, generalized headache, fatigue, lymphadenopathy, back pain, and myalgia [4,5,6,7,8,9]. The most noticeable clinical sign is a skin rash that appears up to three days after the fever has subsided. Frequently, its onset progresses from maculopapules to vesicles, pustules, and then crusts. It is frequently observed on the face and quickly appears in a centrifugal distribution on the body, including extremities, but can be observed throughout the entire body in more severe cases [9,10]. However, monkeypox’s overall clinical presentation is similar to, but less severe, than smallpox [9]. Historically, the largest outbreak of monkeypox was reported in Nigeria in 2017 with 197 suspected cases and 68 confirmed cases [9], and few cases were reported in the United States, transmitted from infected native prairie dogs that were housed with infected exotic pets imported from Africa [11], as well as the United Kingdom (UK), where patients were travelers who had returned from Nigeria [12]. On 13 May 2022, WHO was notified of two laboratory-confirmed monkeypox cases and one possible case in the UK [12]. On 15 May 2022, four additional laboratory-confirmed cases were reported amongst Sexual Health Services attendees presenting with a vesicular rash illness in men who have sex with men (MSM) in the UK [13]. In addition to the limited cross-protection of childhood smallpox vaccines against monkeypox among adults aged 40–50 years and above, the younger population from non-endemic regions also has lower immunity to monkeypox infection. However, the approved vaccine and treatment, Modified Vaccinia Ankara-Bavarian Nordic (MVA-BN) and tecovirimat, respectively, remain widely unavailable [14].

The rise in incidence of human monkeypox emphasizes the importance of preventive measures, and early screening and detection. However, a report by the World Health Organization (WHO) showed that one of the challenges faced in preventing the reemergence of monkeypox was a lack of knowledge of monkeypox [13]. In Saudi Arabia, the first case of monkeypox was identified in the middle of July 2022 in the country’s capital, Riyadh, in a person with a history of international travel from Europe [15]. Hence, to reduce the likelihood of the monkeypox virus spreading across the country, the general public needs to be informed about the disease, understand its hazards, and know how to protect themselves. To explore the level of knowledge regarding monkeypox and provide baseline information, this study, therefore, aimed to assess the knowledge of monkeypox among the general population of Saudi Arabia.

## 2. Materials and Methods

### 2.1. Study Design

We conducted a cross-sectional web-based study among the general Saudi population from 25 May 2022 to 15 July 2022. All Saudi people aged over 18 years were eligible to be included in this study. We used the Raosoft sample size calculator to calculate the minimum required sample size. By assuming that 50% of the population would have good knowledge with a 5% margin of error and a confidence interval of 95%, the minimum sample size of 385 was determined.

### 2.2. Data Collection Tool

We used an anonymous online questionnaire for data collection. The questionnaire had two sections: The first section contained information on sociodemographic characteristics, including current location, gender, age, education, employment, and monthly income. It also included information about chronic diseases and vaccination history (vaccination against COVID-19 and childhood vaccine as per EPI and national guidelines). The second section consisted of 23 multiple-choice questions assessing knowledge of monkeypox designed based on previous studies [1,16,17,18,19] and existing facts from the United States Centers for Disease Control and Prevention (CDC) [20]. Respondents had to choose answers to each question from “Yes”, “No”, and “I don’t know”. The questionnaire was pre-tested on 15 participants from the general population and reviewed by three experts in preventive medicine for checking reliability and clarity. The pilot study results were only used for the improvement of the clarity of questions.

### 2.3. Data Collection Procedure

Participants were asked to fill out a structured questionnaire (see Supplementary Materials) on a specifically designated platform Google Forms link and we distributed it via social media platforms such as WhatsApp, Facebook, and Twitter. Each respondent was requested for their informed consent by clicking the consent statement before attempting to fill out the responses. “I do hereby, after reading the purposes of the study, participate in the survey providing my information by responding questions sensibly and voluntarily,” was the informed consent statement provided to the respondents. After finishing the survey, they tapped the “submit” icon to send it to our data collection platform. It was mandatory to answer all the items to submit a valid response.

### 2.4. Study Variables

The response variable was Saudi population’s knowledge of monkeypox, which was assessed through 23 questions with “Yes, No, and I don’t know” responses. The knowledge score ranged from 0 (lowest) to 23 (highest).

The explanatory variables were: age, which was categorized into 18–25 years, 26–45 years, and more than 46 years; gender, which was dichotomous (male or female); marital status (single or married); participants’ residency region of Saudi Arabia (middle, southern, northern, western, and eastern); and urban or rural area. For assessing obesity, we obtained respondents’ self-reported weight and height, then BMI was calculated as individuals’ weight (in kilograms) divided by the square of their height (meters) [21]. Furthermore, participants were asked about employment status, healthcare worker job type, presence of chronic diseases, obesity (BMI ≥ 30), smoking status, and monthly income. The income was measured in Saudi Riyal (SAR) and divided into four categories: <3000 SAR; 3000–10,999 SAR; 11,000–15,999 SAR and ≥16,000 SAR. Education was grouped into three levels: high school and below; bachelor and postgraduate degrees. Participants were asked if they had completed all their childhood vaccines. To assess the source of information about monkeypox, participants had to choose all sources applicable to their responses from TV and radio, social media, healthcare providers, family or friends, books, and research and articles. Participants were asked if they thought monkeypox would affect social and economic life like the COVID-19 pandemic or if it was a conspiracy or bioterrorism, and then asked about symptoms caused by monkeypox.

### 2.5. Data Analysis

We used IBM SPSS Statistics, Version 26 (IBM Corp, Armonk, NY, USA) for statistical analyses. Continuous variables were presented as mean ± SD (standard deviation), and categorical variables were expressed in frequency (percentage). The data (especially knowledge scores) were found to be normally distributed to use mean and SD, checked by Shapiro–Wilk test. Further, the knowledge scores were dichotomized into two categories: low and high. We used the mean score of 14 ± 5 as a cut-off point; a mean score > 14 was considered high, while 14 and less was considered low. Pearson’s Chi-square test was used to compare response variables and explanatory variables. *p*-value was set at <0.05 for statistical significance.

## 3. Results

Of the 498 responses recorded, 480 responses met the eligibility criteria for inclusion in the final analysis. The participants’ ages ranged from 18 to 65 years, with a mean age of 30.6 ± 10.7 years. The majority of respondents were aged 18–25 years and 26–45 years (45% and 44.4%, respectively). More than half of them were female (58.8%), and not married (58.8%). Most were urban residents (86.9%), had bachelor’s degrees (64.4%), were unemployed (50.6%), earned less than 3000 SAR per month (43.1%), and had no chronic diseases (85.6%). Regarding vaccination history, 55.1% of the participants had completed childhood vaccination, while 44.9% had two or more doses of COVID-19 vaccines (Table 1).

Table 2 shows participants’ responses to questions assessing their knowledge of monkeypox. The majority of participants were aware that it was an infectious disease and did not view it as a conspiracy or act of bioterrorism. Though most knew that monkeypox was also contagious viral (70%), they did not think it would economically and socially be disruptive as the COVID-19 pandemic. Of all participants, 51.9% knew that monkeypox was available even before, 53.8% correctly responded that monkeypox was not sexually transmissible, 55% correctly knew that chickenpox and monkeypox were different diseases, 61.3% correctly knew that monkeypox was not common in the middle east countries, and 65.6% knew the correct common location of monkeypox (Western and Central Africa). In addition, most of the participants knew that no cases were reported in Saudi Arabia (79.4%) and that cases were increasing in the USA and Europe (65.6%). Even if the majority knew that monkeypox could be transmitted from humans to humans (84.4%), the minority correctly knew that it could be transmitted to humans through bites and scratches from infected animals (41.3%). However, most could correctly answer that it is transmitted through droplets (60%) and bodily fluid (53.8%). When assessed about monkeypox symptoms knowledge, most (80%) could identify skin rash as a symptom, but less (53.8%) correctly knew that it also has symptoms similar to the flu. Regarding preventive measures, 63% correctly knew that hand sanitizers and face masks were preventive measures that could be used. The minority correctly knew that no monkeypox vaccine is available in Saudi Arabia (41.9%), and that a smallpox vaccine can be used for vaccination against monkeypox (43.1%). However, even less (34.4%) correctly knew that their childhood chickenpox could not protect them from monkeypox.

Table 3 shows the relationship between the knowledge score and the socio-demographics of respondents. The mean score was 13.81 with a standard deviation of ± 5.44. Therefore, we used 14 ± 5 as the cut point, and a score of more than 14 was considered high knowledge, while 14 and less was considered low knowledge. Age (*p* < 0.01), marital status (*p* < 0.01), living region (*p* < 0.01) and residency areas (*p* = 0.02), education (*p* = 0.000), employment (*p* < 0.01), being a healthcare worker (*p* < 0.01), income (*p* = 0.000), and smoking (*p* < 0.01) had a significant correlation with knowledge about monkeypox. Age was positively correlated with knowledge score, which increased with age, with most (58.8%) participants aged 45 and above scoring high (≥15). High knowledge scores were more likely to be among married (54.4%) respondents, from the middle region (58.8%), with postgraduate degrees (71.4%), employed (60.8%), healthcare workers (75%), ≥16,000 SAR monthly earners (79.3%), and smokers (64%). However, though urban residency (50.4%) was relatively correlated with more respondents with higher scores than rural residents, residential areas (urban and rural) generally correlated with low knowledge scores (≤14). There was a significant correlation between knowledge scores and the expected impact of monkeypox on social and economic lives (*p* = 0.02), with low scores among more respondents who expected it to be as impactful as COVID-19 than those who did not (61.5% vs. 49.6%). Respondents who thought monkeypox was a chronic disease (100%), immune disease (64.7%), hereditary (100%), or metabolic (56.8%) had significantly (*p* < 0.01) low knowledge scores compared to those who thought that monkeypox was infectious (53.4) or inflammatory (54.5%).

Overall, social media (75.0%) was the most frequently reported source of information among the study participants followed by TV and radio (45.6%), family or friend (15.6%), and healthcare provider (13.8%). We found a significant relationship between knowledge score and sources of information about monkeypox (*p* < 0.01), shown in Table 4. The majority of respondents with high knowledge reported healthcare providers (86.4%), research articles (78.6%), and books (66.7%) as their information sources compared to those who used TV and radio (45.2%), social media (48.3%), and family or friends (52%).

## 4. Discussion

As more and more cases of monkeypox are rising in different countries, the Saudi government and Saudi citizens should be ready for the outbreak containment. While public health and healthcare officials are responsible for establishing strategies for prevention, control, and treatment of outbreaks, public engagement is vital for successful measures. Assessment of knowledge about monkeypox among the Saudi population would help set a foundation for measures to educate the public about monkeypox, and engage it in control, prevention, and treatment measures; thus, successfully controlling and eliminating the monkeypox outbreak. Therefore, our study evaluated knowledge of monkeypox among the general population of Saudi Arabia.

The results showed that more than half (52%) of the respondents had low knowledge about monkeypox infection. Saudi Arabia is not an endemic area for monkeypox infection, which is prevalent in the tropical rainforest region. Therefore, the Saudi population is not used to it, which might be why most respondents scored lower. These findings align with other studies which reported lower knowledge about uncommon outbreaks in the study areas [1,17,22].

Around half of the participants knew that monkeypox has been around before, was different from chickenpox, and was not sexually transmissible. This is more than expected considering the rarity of monkeypox cases in Saudi Arabia and even low knowledge reported by a previous study among general practitioners supposed to have good knowledge [17]. A study conducted in Indonesia found that general practitioners had low knowledge about monkeypox and only 10% could correctly answer 80% of questions about monkeypox [1]. Another recent study conducted in Italy found that medical professionals’ knowledge of monkeypox was relatively unsatisfactory, with significant knowledge gaps in this subject [23]. The effect of a rarity on knowledge is supported by the majority who reported that monkeypox is not common in the Middle Eastern region and over three-quarters who did not hear of cases in Saudi Arabia. Similarly, other studies conducted in Indonesia showed that other rare outbreaks were associated with low knowledge among healthcare professionals, while they had high knowledge about Indonesia’s endemic outbreaks [22,24]. On the other hand, we found that around two-thirds of respondents knew about the current emergence of monkeypox in different countries, the vast majority knew about skin rash as a symptom, and knew about human-to-human transmission through droplets and bodily fluid. This may be a result of the current digital era, with its rapid information sharing and the aid of the internet, which facilitates the rapid dissemination of information regarding monkeypox by Saudi public health authorities. Studies have shown that easy internet access boosted easy access to health information among the general public [25]. Another study carried out in India about the recent Zika virus outbreak also showed that the internet was the main source of information about the Zika virus, which is not common in India [26,27]. However, the internet could be the main source of misinformation and myths, especially about outbreak treatments, by coming up with other incorrect alternatives or sometimes options endangering the audience. Some of the myths about monkeypox are: monkeypox is sexually transmissible, is transmitted by only monkeys, is a conspiracy, has a secret treatment, and was made in a laboratory, to name a few, prompting the WHO to publish a Q&A (Questions and Answers) [26]. These myths are similar to COVID-19-related myths, which is not surprising since monkeypox reemergence coincided with the COVID-19 pandemic and both are viral infections [26,27].

Myth believers are more likely to have low knowledge levels, which is demonstrated by our findings. Participants who expected monkeypox to be as socially and economically impactful as COVID-19 had low knowledge scores compared to those who did not expect it to be like COVID-19. Moreover, more participants who could not identify what kind of disease monkeypox was scored lower than those who correctly identified it as infectious. This indicates that there might be an influence from conspiracies or myths. In line with this hypothesis, respondents who thought that monkeypox was a conspiracy or terrorist attack also scored lower, though statistically insignificant. Sallam et al. reported that conspiracy beliefs about emerging virus infections were widely prevalent among Jordanian students. They also showed that older age, females, and affiliation to non-medical schools/faculties were significantly associated with harboring higher levels of conspiracy beliefs regarding emerging virus infections [28].

While the presence of monkeypox virus in the semen was reported, sexual transmission is still being investigated [13,26]. Though rodents can transmit monkeypox, only 46.3% of respondents knew that it is transmitted not only by monkeys, while 40.6% were unsure. The 2003 USA monkeypox outbreak was attributed to rodents imported from Ghana as pets [8]. Trustworthy information might have been shadowed by misinformation and might be the reason that less than 40% of our study respondents knew that no specific treatment exists for monkeypox, and that smallpox vaccine could not protect against monkeypox. Another reason might be the combination of the rarity of monkeypox in Saudi Arabia and the more technical nature of treatment procedures for outbreaks, including monkeypox. Though the childhood smallpox vaccine cannot protect adults from monkeypox, the smallpox vaccine for adults has shown up to 85% protection against monkeypox. Harapan et al. reported that 71.9% of physicians were willing to take smallpox vaccines to be protected against monkeypox [27].

Regarding the assessment of assessing the relationship between knowledge about monkeypox and the socio-demographics of respondents, we found that age significantly correlated with high knowledge scores, which increased with age. Monkeypox has been around for over 40 years, which might explain higher knowledge scores in those participants aged over 45 years old [2]. Moreover, the older they are, the more likely they are to be married and the more knowledge they have, as we found that married respondents also scored higher than single ones. In contrast, a study on general practitioners found that younger ones had higher knowledge than the old ones, which might be explained by more familiarity of younger generations with the internet and easier access to updated information [1]. Married couples have extra protective responsibilities towards their family members, especially children, which makes them always pay more attention to health information and news.

Respondents with higher education levels, employed, healthcare workers and high-income earners had higher knowledge scores, which is expected since healthcare, workers who have good knowledge, tend to be also highly educated and high-income earners. In addition, highly educated people tend to be employed and have better-paying jobs that enable them to have access to accurate information from scientists and other professionals [29]. People in this category also tend to be involved in publications, read scientific publications, and equip themselves with better knowledge and skills. In line with our findings, a study conducted assessing knowledge of dengue fever infection among the general population showed that high income and high education levels were associated with up to two times better knowledge [22]. The influence of information sources on the level of knowledge was also indicated by our study results that showed higher knowledge scores among respondents who sourced their information about monkeypox from healthcare professionals, research articles, and books compared to those who sourced information from the media, family and friends.

Residential location can have also an impact on knowledge, especially nowadays when there is a developed internet-based spread of information. Developed, mostly urban areas might influence access to information and, consequently, affect knowledge. This is supported by our findings that respondents from urban areas with high knowledge scores had more than those with high knowledge scores from rural areas. These findings also align with a study conducted in Indonesia about monkeypox involving general practitioners [1], and about dengue fever [22].

This study this study had some limitations, such as cross-sectional design which limited identification of the association between exposure and outcomes due to measuring both simultaneously. This study was prone to selection bias due to its being online and with convenience sampling. Our sample was small, which might not be generalizable. Face to face interview-based longitudinal studies with randomization and larger samples are recommended and could confirm our results.

## 5. Conclusions

We found that overall knowledge of monkeypox infection was poor among the general population in Saudi Arabia. Better knowledge was associated with being older, employed, married, a healthcare worker, having a high income, and having a higher education. This indicates that those with access to trustworthy information are highly knowledgeable. Higher knowledge scores were more common among respondents who sourced their information about monkeypox from healthcare professionals, research articles, and books compared to those who sourced information from the media, family, and friends. These findings highlight the urgent need for public education on monkeypox, which is still not reported in Saudi Arabia, to raise awareness and engage the public ahead of the outbreak. Healthcare facilities should also be involved in preparations to contain monkeypox and get familiar with it through campaigns and consultancy from health officials from endemic regions worldwide.

The monkeypox outbreak is spreading internationally, and Saudi health authorities are recommended to initiate preventive, control, and treatment measures, including the introduction of the MVA-BN vaccine to protect relatively low-immunized and high-risk groups, and tecovirimat to treat suspected cases. Since monkeypox can spread from animals to people, animal health officials should also be involved in all steps that are taken.

## Figures and Tables

**Table 1:** Socio-demographics characteristics of the study respondents (*n* = 480).

Variables		N (%)
Age (Years)	18–25	216 (45)
	26–45	213 (44.4)
	>45	51 (10.5)
Gender	Female	282 (55.8)
	Male	198 (41.3)
Marital status	Single	282 (58.8)
	Married	198 (41.3)
Region of Saudi Arabia	Middle	102 (21.3)
	Southern	174 (36.3)
	Northern	78 (16.3)
	Western	63 (13.1)
	Eastern	63 (13.1)
Residency	Rural	63 (13.1)
	Urban	417 (86.9)
Education level	High school and below	66 (13.8)
	Bachelor’s degree	309 (64.4)
	Postgraduate degree	105 (21.9)
Employment	No	243 (50.6)
	Yes	237 (49.4)
Healthcare worker	No	360 (75)
	Yes	120 (25)
Monthly income	<3000 SAR/month	207 (43.1)
	3000–10,999 SAR/month	117 (24.4)
	11,000–15,999 SAR/month	69 (14.4)
	≥16,000 SAR/month	87 (18.1)
Chronic disease	Yes	69 (14.37)
	No	411 (85.63)
	Obesity (BMI ≥ 30)	84 (34.1)
	Smoking	75 (30.5)
Vaccination history	Two or more COVID-19 vaccine shots	357 (44.9)
	Childhood vaccination complete	438 (55.1)
Do you think monkeypox will affect social and economic life like the COVID-19 pandemic?	No	363 (75.6)
	Yes	117 (24.4)
In your opinion, is monkeypox a conspiracy or bioterrorism?	No	369 (76.9)
	Yes	111 (23.1)

SAR = Saudi Riyal (1 SAR = 3.75 USD).

**Table 2:** Responses to questions assessing participants’ knowledge about monkeypox virus.

Statements	Answers	N (%)
What kind of disease does monkeypox cause?	Chronic disease	21 (4.4)
	Immune disease	51 (10.6)
	Infectious disease *	348 (72.5)
	Hereditary	9 (1.9)
	inflammation	33 (6.9)
	Metabolic	18 (3.8)
Monkeypox is a new infection that appeared this year 2022.	No *	249 (51.9)
	Yes	183 (38.1)
	I do not know	48 (10)
Monkeypox is a sexually transmitted disease.	No *	258 (53.8)
	Yes	162 (33.8)
	I do not know	60 (12.5)
Chickenpox and monkeypox are the same disease.	No *	264 (55)
	Yes	69 (14.4)
	I do not know	147 (30.6)
Monkeypox is common in Middle Eastern countries.	No *	294 (61.3)
	Yes	57 (11.9)
	I do not know	129 (26.9)
Monkeypox is common in West and Central African countries.	No	60 (12.5)
	Yes *	315 (65.6)
	I do not know	105 (21.9)
There are many cases recorded in Saudi Arabia.	No *	381 (79.4)
	Yes	21 (4.4)
	I do not know	78 (16.3)
Monkeypox cases are increasing in the USA and Europe.	No	51 (10.6)
	Yes *	315 (65.6)
	I do not know	114 (23.8)
Monkeypox is a contagious viral disease.	No	81 (16.9)
	Yes *	336 (70)
	I do not know	63 (13.1)
Monkeypox is a contagious bacterial disease.	No *	246 (51.2)
	Yes	156 (32.5)
	I do not know	78 (16.3)
Monkeypox is easily transmitted from one person to another.	No *	186 (38.8)
	Yes	201 (41.9)
	I do not know	93 (19.4)
Monkeypox is transmitted to humans through the bites and scratches from infected animals.	No	132 (27.5)
	Yes *	198 (41.3)
	I do not know	150 (31.3)
People with monkeypox can transmit the disease to others (the disease is transmitted between humans).	No	27 (5.6)
	Yes *	405 (84.4)
	I do not know	48 (10)
Monkeypox is spread by droplets (coughing and sneezing).	No	108(22.5)
	Yes *	291 (60.6)
	I do not know	81 (16.9)
The first symptoms of monkeypox are similar to the flu.	No	87 (18.1)
	Yes *	258 (53.8)
	I do not know	135 (28.1)
Skin rash is a symptom of monkeypox.	No	33 (6.9)
	Yes *	384 (80)
	I do not know	63 (13.1)
Monkeypox only affects males.	No *	378 (78.8)
	Yes	30 (6.3)
	I do not know	72 (15)
Hand sanitizers and face masks are important in preventing monkeypox.	No	78 (16.3)
	Yes *	306 (63.7)
	I do not know	96 (20)
There is a special treatment for monkeypox.	No *	189 (39.4)
	Yes	147 (30.6)
	I do not know	144 (30)
Monkeypox is spread through bodily fluids.	No	78 (16.3)
	Yes *	258 (53.8)
	I do not know	144 (30)
There is a monkeypox vaccine available in Saudi Arabia.	No *	201 (41.9)
	Yes	129 (26.9)
	I do not know	150 (31.3)
The chickenpox vaccine I got in childhood protects me from monkeypox.	No *	165 (34.4)
	Yes	138 (28.7)
	I do not know	177 (36.9)
There is a smallpox vaccine that can be used for monkeypox.	No	90 (18.8)
	Yes *	207 (43.1)
	I do not know	183 (38.1)

* Correct answer.

**Table 3:** Relationship between the knowledge score and the socio-demographics of respondents. (*n* = 480).

Variables	Categories	Knowledge Level		*p*-Value
Overall knowledge score		Low (≤14) N (%) 252 (52)	High (>14) N (%) 228 (48)	
Age (Years)	18–25	132 (61.1)	84 (38.9)	<0.01 *
	26–45	99 (46.5)	114 (53.5)	
	>45	21 (41.2)	30 (58.8)	
Gender	Female	153 (54.3)	129 (45.7)	0.35
	Male	99 (50)	99 (50)	
Marital status	Single	162 (57.4)	120 (42.6)	<0.01 *
	Married	90 (45.5)	108 (54.5)	
Region of Saudi Arabia	Middle	42 (41.2)	60 (58.8)	<0.01 *
	Southern	90 (51.7)	84 (48.3)	
	Northern	57 (73.1)	21 (26.9)	
	Western	33 (52.4)	30 (47.6)	
	Eastern	30 (47.6)	33 (52.4)	
Residency	Rural	42 (66.7)	21 (33.3)	<0.01 *
	Urban	210 (50.4)	207 (49.6)	
Education level	High school and below	42 (63.6)	24 (36.4)	<0.01 *
	Bachelor’s degree	180 (58.3)	129 (41.7)	
	Postgraduate degree	30 (28.6)	75 (71.4)	
Employee	No	159 (65.4)	84 (34.6)	<0.01 *
	Yes	93 (39.2)	144 (60.8)	
Healthcare worker	No	222 (61.7)	138 (38.3)	<0.01 *
	Yes	30 (25)	90 (75)	
Monthly income	<3000 SAR/month	132 (63.8)	75 (36.2)	<0.01 *
	3000–10,999	63 (53.8)	54 (46.2)	
	11,000–15,999	39 (56.5)	30 (43.5)	
	≥16,000 SAR/month	18 (20.7)	69 (79.3)	
Obesity (BMI ≥30)	No	201 (50.8)	195 (49.2)	0.09
	Yes	51 (60.7)	33 (39.3)	
Smoking	No	225 (55.6)	180 (44.4)	<0.01 *
	Yes	27 (36)	48 (64)	
Two or more COVID-19 vaccine shots	No	41 (33.4)	82 (66.6)	0.06
	Yes	153 (42.9)	204 (57.1)	
Childhood vaccination complete	No	27(64.3)	15(35.7)	0.10
	Yes	225 (51.4)	213 (48.6)	
Do you think monkeypox will affect social and economic life like the COVID-19 pandemic?	No	180 (49.6)	183 (50)	0.02 *
	Yes	72 (61.5)	45 (38.5)	
In your opinion, is monkeypox a conspiracy or a terrorist act?	No	189 (51.2)	180 (48.8)	0.31
	Yes	63 (56.8)	48 (43.2)	

* Statistically significant (*p* < 0.05).

**Table 4:** Relationship between knowledge score and sources of information about monkeypox.

Source of Information	Total, *n* (%)	Knowledge Score		*p*-Value
		Low, *n* (%)	High, *n* (%)	
		252 (52)	228 (48)	
TV and radio	219 (45.6)	120 (54.8)	99 (45.2)	
Social media	360 (75.0)	186 (51.7)	174 (48.3)	
Healthcare provider	66 (13.8)	9 (13.6)	57 (86.4)	<0.01 *
Family or friend	75 (15.6)	36 (48)	39 (52)	
Books	18 (3.75)	6 (33.3)	12 (66.7)	
Research articles	42 (8.8)	9 (21.4)	33 (78.6)	

* Statistically significant (*p* < 0.05).

## Data Availability

Not applicable.

## Supplementary Materials

The following are available online at https://www.mdpi.com/article/10.3390/pathogens11080904/s1, Survey: Assessment of Knowledge of Monkeypox Viral Infection among the General Population in Saudi Arabia.
