# Supplementary material for: Assessment of Knowledge of Monkeypox Viral Infection among the General Population in Saudi Arabia
Source: Pathogens. 2022 Aug 11;11(8):904. doi: 10.3390/pathogens11080904 (PMC9414752; doi:10.3390/pathogens11080904)
Supplement: Supplementary file 1 [file pathogens-11-00904-s001.zip › pathogens-1830432-supplementary.pdf]

Assessment of Knowledge of Monkeypox Viral Infection among the General Population in Saudi Arabia

Survey

Consent question:

- I am a Saudi person, and my age is over 18 years old. And I voluntarily agree to be a part of this research.
  - ✓ Yes
  - ✓ No

Socio-demographics questions:

- Age in years …..
- Gender
  - ✓ Male
  - ✓ Female
- Marital status
  - ✓ Married
  - ✓ Single
- Which region of Saudi Arabia you are from?
  - ✓ Central
  - ✓ Western
  - ✓ Northern
  - ✓ Southern
  - ✓ Eastern
- Where do you live?
  - ✓ Ruler
  - ✓ Urban
- What is your last education level?
  - ✓ High school and below
  - ✓ Postgraduate degree
- What is your monthly income? State it number….
- Do you have any chronic diseases?
  - ✓ Yes
  - ✓ No
- Are you an employee?
  - ✓ Yes
  - ✓ No
- Are you a healthcare worker?
  - ✓ Yes
  - ✓ No
- Your weight in kg …
- Your height in cm…
- Are you a smoker?
  - ✓ Yes
  - ✓ No
- Have you gotten all COVID-19 vaccine shots?
  - ✓ Yes

- ✓ No
- Did you complete your childhood vaccination?
- ✓ Yes
- ✓ No
- Do you think monkeypox will affect social and economic life like the COVID-19 pandemic?
- ✓ Yes
- ✓ No
- In your opinion, is monkeypox a conspiracy or bioterrorism?
- ✓ Yes
- ✓ No

__Knowledge about monkeypox questions:__

- What kind of disease does monkeypox cause?
- ✓ Immune disease
- ✓ Infectious disease
- ✓ Hereditary
- ✓ inflammation
- ✓ Metabolic

- Monkeypox is a new infection that appeared this year 2022
- ✓ Yes
- ✓ No
- ✓ I don't know

- Monkeypox is a sexually transmitted disease.
- ✓ Yes
- ✓ No
- ✓ I don't know

- Chickenpox and monkeypox are the same disease.
- ✓ Yes
- ✓ No
- ✓ I don't know

- Monkeypox is common in Middle Eastern countries.
- ✓ Yes
- ✓ No
- ✓ I don't know

- Monkeypox is common in West and Central African countries.
- ✓ Yes
- ✓ No
- ✓ I don't know

- There are many cases recorded in Saudi Arabia.
- ✓ Yes
- ✓ No
- ✓ I don't know

- Monkeypox cases are increasing in the USA and Europe.
- ✓ Yes
- ✓ No
- ✓ I don't know

- Monkeypox is a contagious viral disease.
- ✓ Yes
- ✓ No
- ✓ I don’t know

- Monkeypox is a contagious bacterial disease.
- ✓ Yes
- ✓ No
- ✓ I don’t know

- Monkeypox is easily transmitted from one person to another.
- ✓ Yes
- ✓ No
- I don’t know

- Monkeypox is transmitted to humans through the bites and scratches from infected animals.
- ✓ Yes
- ✓ No
- ✓ I don’t know

- People with monkeypox can transmit the disease to others (the disease is transmitted between humans).
- ✓ Yes
- ✓ No
- ✓ I don’t know

- Monkeypox is spread by droplets (coughing and sneezing).
- ✓ Yes
- ✓ No
- ✓ I don’t know

- The first symptoms of monkeypox are similar to the flu.
- ✓ Yes
- ✓ No
- ✓ I don’t know

- Skin rash is a symptom of monkeypox.
- ✓ Yes
- ✓ No
- ✓ I don’t know

- Monkeypox only affects males.
- ✓ Yes
- ✓ No
- ✓ I don’t know

- Hand sanitizers and face masks are important in preventing monkeypox.
- ✓ Yes
- ✓ No
- ✓ I don’t know

- There is a special treatment for monkeypox.
- ✓ Yes
- ✓ No

- ✓ I don’t know

- Monkeypox is spread through bodily fluids.
  - ✓ Yes
  - ✓ No
  - ✓ I don’t know

- There is a monkeypox vaccine available in Saudi Arabia.
  - ✓ Yes
  - ✓ No
  - ✓ I don’t know

- The chickenpox vaccine I got in childhood protects me from monkeypox.
  - ✓ Yes
  - ✓ No
  - ✓ I don’t know

- There is a smallpox vaccine that can be used for monkeypox.
  - ✓ Yes
  - ✓ No
  - ✓ I don’t know

What are your main sources of your current information about Monkeypox? You may choose more than answer

- ✓ TV and radio
- ✓ Social media
- ✓ Healthcare provider
- ✓ Family or friend
- ✓ Books
- ✓ Research articles
